# Blade Vibration Difference-Based Circumferential Fourier Fitting Algorithm for Synchronous Vibration Parameter Identification of Rotation Blades

**DOI:** 10.3390/s24248083

**Published:** 2024-12-18

**Authors:** Zhenfang Fan, Hongkun Li, Jinying Huang, Siyuan Liu

**Affiliations:** 1 School of Mechanical Engineering, North University of China, Taiyuan 030051, China; jyhuang@nuc.edu.cn; 2 School of Mechanical Engineering, Dalian University of Technology, Dalian 116024, China; lihk@dlut.edu.cn; 3 School of Data Science and Technology, North University of China, Taiyuan 030051, China; 20230120@nuc.edu.cn

**Keywords:** blade tip timing, blade vibration difference, circumferential Fourier fitting, synchronous vibration parameter identification

## Abstract

Blades are the core components of rotating machinery, and the blade vibration status directly impacts the working efficiency and safe operation of the equipment. The blade tip timing (BTT) technique provides a solution for blade vibration monitoring and is currently a prominent topic in research on blade vibration issues. Nevertheless, the non-stationary factors present in actual engineering applications introduce inaccuracies in the BTT technique, resulting in blade vibration measurement errors. The theory of blade vibration difference offers a new perspective for high-precision BTT techniques. This paper optimizes the traditional circumferential Fourier fitting (CFF) algorithm. According to the blade departure time measurement mechanism, four sets of BTT signals are obtained by two probes, six sets of blade vibration differences are established, and, then, a blade vibration difference-based circumferential Fourier fitting (BVD-CFF) algorithm for blade synchronous vibration parameter identification is proposed. Simulation studies demonstrate that the BVD-CFF algorithm exhibits superior anti-noise performance. Moreover, experimental investigations on a high-speed rotation blade vibration test rig and a large-scale centrifugal compressor test rig display that the engine order of blade synchronous vibrations obtained by the BVD-CFF algorithm are essentially the same as those obtained by the strain gauge method.

## 1. Introduction

Blades are the core component of rotating machinery working on the gas medium, which are widely used in aviation, aerospace, petroleum, chemical and other industrial fields [1]. The development of major equipment is gradually shifting towards high-load, high-speed, and large-scale. Nevertheless, rotation blades operate in complex and harsh environments for extended periods of time, and rotation effects, unsteady flow loads, and alternating stresses generated by dynamic and static components result in blade vibration [2]. Blade vibration (BV) can induce localized stress concentration on the blade surface, thereby leading to typical faults such as fatigue, wear, crack, fracture, and so on [3,4]. Blade failures directly affect the health and stable operation of the equipment, not only necessitating shutdowns and maintenance that disrupt production schedules but also posing a risk for safety accidents [5]. Hence, it is imperative to conduct the vibration monitoring and operational status evaluation of rotation blades for the advancement and safe functioning of major equipment [6].

The existing technologies for vibration measurements of rotation blades primarily include the strain gauge (SG) method [7] and the blade tip timing (BTT) technique [8]. The former involves attaching strain gauges to the blades, and it has limitations such as a restricted number of measurement points and a short lifespan [9], making it more suitable for product development and testing purposes. On the other hand, the BTT technique provides an effective solution for monitoring blade vibration by sensing the lead or lag time of blade arrival at the probe. The BTT technique addresses challenges encountered in practical applications of the SG method, offering distinct advantages such as continuity, synchronization, and real-time monitoring [10]. Hence, the BTT technique has emerged as a prominent approach for investigating blade vibration within international scientific research institutions.

Currently, scholars worldwide have conducted extensive research on the BTT technique and have achieved significant results [11]. However, in practical applications, the extreme operating environments and complex exciting force components induce the non-stationary factors, such as speed fluctuations, probe vibration, acquisition system resolution errors, and so on, still contribute to the “uncertainty” problem in the BTT technique, such as blade vibration measurement errors, blade vibration parameter identification errors, and so on [12]. The traditional methods assume that the rotation speed is constant when the rotor completes one revolution [13,14] and ignores the influence of speed fluctuation, which leads to the reconstruction error of the expected arrival time of the blade. To mitigate blade vibration measurement errors caused by speed fluctuations, scholars have conducted a significant amount of research on algorithms for blade vibration calculations. Fan et al. [15] proposed to use the interpolation of key-phase signals to determine the speed changes in each revolution. Subsequently, Ren et al. [16] analyzed the calculation errors caused by speed fluctuation and proposed the error correction BTT method. Nevertheless, the installation of the key-phase acquisition system presents challenges, making it difficult to accurately calculate blade vibration with key-phase signals. Furthermore, the uncertainty associated with key-phase testing also contributes to errors in blade vibration measurements [17].

In recent years, to enhance the reliability and stability of the BTT technique, the approach of extracting virtual key-phase signals from BTT signals has emerged as a hotspot in the BTT technique. Russhard [18] derived the blade expected arrival time through linear fitting BTT signals. Subsequently, Chen et al. [19] enhanced the algorithm and proposed the optimal solution theory for linear fitting, using it as a reference key phase for calculating blade vibration. He et al. [20] also investigated the error transfer law of the key-phase method and put forward a reconstruction method for virtual key-phase signals. In conclusion, the precise acquisition of the key-phase signals and the blade expected arrival time is crucial for blade vibration measurement. The key-phase-free methods solve the installation problem of the key-phase acquisition system and the measurement errors of blade vibration induced by the key-phase test error. But, extracting the key-phase signals or blade expected arrival time through function fitting will introduce the fitting errors, leading to the “uncertainty” problem in the BTT technique [21].

Blade vibration parameter identification is also significant for the state evaluation of blade vibration [22]. The principle of BTT techniques exhibits that the sampling frequency of a single probe is approximately equal to the rotation frequency. In actual applications, the blade vibration frequency is obviously higher than the rotation frequency, and the blade vibration signal obtained by the BTT technique does not comply with the Nyquist sampling theorem for frequency identification [23]. For the past few years, scholars have carried out extensive explorations on the parameter identification algorithms of blade vibration, taking into account probe layout [24], blade vibration type [25], and operating conditions [26]. The identification algorithms of blade synchronous vibration parameters mainly include the single-parameter method [27,28], the two-parameter plot (2PP) method [29,30], the autoregressive (AR) method [31,32], and the circumferential Fourier fitting (CFF) method [33,34]. The single-parameter method is based on the response model of the blade vibration, which can be either single-frequency or multi-frequency. The nonlinear least square method is utilized to fit the blade vibration waveform and obtain the parameters of blade vibration. Nevertheless, the accuracy of the single-parameter method depends on the quality of the blade vibration signal, and the fitting efficiency cannot meet the requirements of real-time processing in the BTT technique. The vibration waveforms measured by two probes are elliptically fitted with the 2PP method, and, then, the blade vibration parameters are calculated according to the elliptic coefficient. The 2PP method is not robust enough to identify actual blade vibration parameters with a high signal-noise-ratio (SNR). The AR method has strict requirements regarding the number and layout of probes. The CFF method considers the blade single-frequency or multi-frequency vibration and utilizes the least square method to calculate the blade synchronous vibration parameters. The more frequency components of blade vibration, the greater number of probes required by the CFF method.

In a previous study [35], the blade vibration difference (BVD) theory was proposed to characterize the blade vibration state using blade vibration difference information. The BVD theory eliminates the computation process of the key-phase signal and the blade expected arrival time, inhibits the test error induced by non-stationary factors, and realizes high-precision measurements of the blade vibration. Based on the BVD theory, this paper improves the traditional CFF algorithm and proposes a blade vibration difference-based circumferential Fourier fitting (BVD-CFF) algorithm for blade synchronous vibration parameter identification. The BVD-CFF algorithm obtained the arrival time and departure time of the rotation blade through two probes, established six sets of blade vibration difference, and identified blade synchronous vibration parameters by the least square method. Compared with the traditional CFF algorithm, the BVD-CFF algorithm reduced the number of probes, overcame the limitations of the probe layout, and its excellent robustness improved the identification accuracy of blade synchronous vibration parameters.

## 2. Blade Vibration Difference Measurement Theory

### 2.1. Blade Vibration Measurement Method

The existing BTT technique obtains the blade vibration information by calculating the lead or lag of the blade reaching the probe. The formula for calculating blade vibration displacement according to the blade expected arrival time is as follows:

(1) $$y_{\left(i,p\right)}=\omega R\left(t_{\left(i,p\right)}-\tau_{\left(i,p\right)}\right)$$

where *y* is the blade vibration displacement, ω is the rotation speed, *R* is the rotation radius of the blade tip, *i* is the blade index, *p* is the probe index, *t* is the actual arrival time, and τ is the expected arrival time.

The algorithm for the blade expected arrival time has been extensively discussed in previous research [11]; thus, it will not be elaborated upon in detail within this paper.

### 2.2. Blade Vibration Difference Measurement Method

The BVD theory describes the blade vibration state with blade vibration difference information, eliminating the key-phase signals and the blade expected arrival times, thereby significantly enhancing the accuracy of the blade vibration measurement. In this study, an approach for measuring blade vibration difference is introduced. Based on (1), the blade vibration displacement obtained by two probes can be expressed as follows:

(2) $$y_{\left(i,1\right)}=\omega R\left(t_{\left(i,1\right)}-\tau_{\left(i,1\right)}\right)$$

(3) $$y_{\left(i,2\right)}=\omega R\left(t_{\left(i,2\right)}-\tau_{\left(i,2\right)}\right)$$

The blade vibration difference between two probes can be calculated as follows:

(4) $$\begin{array}{ll} \Delta y & =y_{\left(i,2\right)}-y_{\left(i,1\right)}\\ & =\omega R\left[\left(t_{\left(i,2\right)}-\tau_{\left(i,2\right)}\right)-\left(t_{\left(i,1\right)}-\tau_{\left(i,1\right)}\right)\right]\\ & =\omega R\left[\left(t_{\left(i,2\right)}-t_{\left(i,1\right)}\right)-\left(\tau_{\left(i,2\right)}-\tau_{\left(i,1\right)}\right)\right]\\ & =\omega R\left(t_{\left(i,2\right)}-t_{\left(i,1\right)}\right)-R\left(\omega \tau_{\left(i,2\right)}-\omega \tau_{\left(i,1\right)}\right) \end{array}$$

where ωτ can be regarded as the rotation radian without blade vibration.

According to the principle of BTT technique, ωτ can be rewritten as follows:

(5) $$\omega \tau_{\left(i,1\right)}=2\pi n+\beta_{1}$$

where *n* is the revolution index, and β is the installation angle of the probe.

Hence, (4) can be reformulated as follows:

(6) $$\begin{array}{ll} \Delta y & =\omega R\left(t_{\left(i,2\right)}-t_{\left(i,1\right)}\right)-R\left(2\pi n+\beta_{2}-2\pi n+\beta_{1}\right)\\ & =\omega R\left(t_{\left(i,2\right)}-t_{\left(i,1\right)}\right)-R\left(\beta_{2}-\beta_{1}\right) \end{array}$$

Ideally, the time that blade rotates one revolution is equal to the time that the rotor rotates one revolution. Therefore, without considering the influence of blade vibration, the rotation speed can be calculated as follows:

(7) $$\omega \left(n\right)=\frac{2\pi }{N_{b}}\sum_{i=1}^{N_{b}}\frac{2}{t_{\left(i,1\right)}(n+1)-t_{\left(i,1\right)}(n)}$$

where $N_{b}$ is number of blades, and $\omega \left(n\right)$ is the average rotation frequency of the rotor.

Equation (6) demonstrates that the sole factor affecting the calculation of blade vibration difference is the blade actual arrival time, which eliminates the reconstruction errors of the key-phase signals and the blade expected arrival time and enhances the precision of the BTT technique.

### 2.3. Blade Tip Timing Technique with Two Probes

The traditional BTT technique captures the blade vibration information by precisely measuring the blade actual arrival time through the probe. Reference [35] demonstrated that the blade departure time also contains valuable information regarding blade vibration. Utilizing the optical fiber probe technique, this paper employs two probes to examine and analyze the blade vibration characteristics. By simultaneously capturing the arrival time and departure time of rotation blades, four sets of BTT signals were obtained by two probes, and, then, six sets of blade vibration differences can be established. The 1st probe was installed at an angle of 0°, while the 2nd probe was installed at an angle of β, as defined in this study. Table 1 presents the installation angles of the probes corresponding to the four sets of BTT signals. Here, γ denotes the rotation angle of the rotor when the blade passes through the BTT probe, commonly referred to as the blade departure angle.

## 3. Blade Synchronous Vibration Parameter Identification Algorithm

### 3.1. Traditional Circumferential Fourier Fit Algorithm

The traditional CFF algorithm based on the blade vibration is named as the BV-CFF algorithm in this paper. The BV-CFF algorithm assumes a sinusoidal mode of blade vibration, and the blade vibration response can be expressed as follows:

(8) $$y\left(t\right)=A\sin(\omega_{b}t+\phi )+C$$

where *A*, $\omega_{b}$, ϕ and *C* are the amplitude, frequency, phase and offset of blade vibration, respectively.

The blade synchronous vibration means that the vibration frequency is an integer multiple of the rotation frequency; thus, the blade vibration frequency can be written as follows:

(9) $$\omega_{b}=N_{\mathrm{EO}}\omega$$

where $N_{\mathrm{EO}}$ is the engine order (EO) of blade vibration.

Thereby, (8) can be rewritten as follows:

(10) $$y\left(t\right)=A\sin(N_{\mathrm{EO}}\omega t+\phi )+C$$

Based on the BTT technique, when the blade reaches the probe, the radian of the rotor is as follows:

(11) $$\omega t=\int_{0}^{t}\omega dt=2\pi n+\beta$$

Moreover, by substituting (11) into (10), the blade vibration displacement can be expressed as follows:

(12) $$y=A\sin(N_{\mathrm{EO}}\beta +\phi )+C$$

According to Table 1, two probes are capable of capturing four sets of BTT signals. When the rotor completes one revolution, the vibration displacement of the blade measured by two probes is as follows:

(13) $$\left\{\begin{array}{l} y_{1}=A\sin\left(\phi \right)+C\\ y_{2}=A\sin\left(N_{\mathrm{EO}}\gamma +\phi \right)+C\\ y_{3}=A\sin\left(N_{\mathrm{EO}}\beta +\phi \right)+C\\ y_{4}=A\sin\left(N_{\mathrm{EO}}\beta +N_{\mathrm{EO}}\gamma +\phi \right)+C \end{array}\right.$$

By solving (13), the blade synchronous vibration parameters *A*, $N_{\mathrm{EO}}$, ϕ and *C* can be determined. However, (13) represents a highly complex nonlinear system that cannot be directly solved. Through trigonometric transformation, the sine term in (13) can be expanded as follows:

(14) $$\left\{\begin{array}{l} y_{1}=A\sin\left(\phi \right)+C\\ y_{2}=A\sin\left(N_{\mathrm{EO}}\gamma \right)\cos\left(\phi \right)+A\cos\left(N_{\mathrm{EO}}\gamma \right)\sin\left(\phi \right)+C\\ y_{3}=A\sin\left(N_{\mathrm{EO}}\beta \right)\cos\left(\phi \right)+A\cos\left(N_{\mathrm{EO}}\beta \right)\sin\left(\phi \right)+C\\ y_{4}=A\sin\left(N_{\mathrm{EO}}\beta +N_{\mathrm{EO}}\gamma \right)\cos\left(\phi \right)+A\cos\left(N_{\mathrm{EO}}\beta +N_{\mathrm{EO}}\gamma \right)\sin\left(\phi \right)+C \end{array}\right.$$

Equation (14) can be written in matrix form as follows:

(15) Y=BX

where

(16) $$Y={\left[\begin{array}{cccc} y_{1} & y_{2} & y_{3} & y_{4} \end{array}\right]}^{T}$$

(17) $$B=\left[\begin{array}{lll} 0 & 1 & 1\\ \sin\left(N_{\mathrm{EO}}\gamma \right) & \cos\left(N_{\mathrm{EO}}\gamma \right) & 1\\ \sin\left(N_{\mathrm{EO}}\beta \right) & \cos\left(N_{\mathrm{EO}}\beta \right) & 1\\ \sin\left(N_{\mathrm{EO}}\beta +N_{\mathrm{EO}}\gamma \right) & \cos\left(N_{\mathrm{EO}}\beta +N_{\mathrm{EO}}\gamma \right) & 1 \end{array}\right]$$

(18) $$X={\left[\begin{array}{ccc} x_{1} & x_{2} & x_{3} \end{array}\right]}^{T}={\left[\begin{array}{ccc} A\cos\left(\phi \right) & A\sin\left(\phi \right) & C \end{array}\right]}^{T}$$

The vector **X** represents the unknown variables, while matrix **B** serves as the coefficient matrix in the context of an overdetermined linear system (15). The vector **X** encompasses the unknowns *A*, ϕ, and *C*, whereas the coefficient matrix incorporates the unknown variable $N_{\mathrm{EO}}$. The EO of blade synchronous vibration was estimated through the EO-search method [36], followed by the calculation of blade vibration parameters.

### 3.2. Blade Vibration Difference-Based Circumferential Fourier Fit Algorithm

Based on (12), the vibration difference between the blade passing through probes installed at angles $\beta_{1}$ and $\beta_{2}$ can be expressed as follows:

(19) $$\begin{array}{ll} \Delta y & =y_{2}-y_{1}\\ & =A\sin(N_{\mathrm{EO}}\beta_{2}+\phi )-A\sin(N_{\mathrm{EO}}\beta_{1}+\phi )\\ & =2A\sin\left(\frac{N_{\mathrm{EO}}\Delta \beta }{2}\right)\sin\left(\frac{N_{\mathrm{EO}}\sum \beta }{2}+\phi +\frac{\pi }{2}\right)\\ & =2A\sin\left(\frac{N_{\mathrm{EO}}\Delta \beta }{2}\right)\cos\left(\frac{N_{\mathrm{EO}}\sum \beta }{2}\right)\cos\left(\phi \right)-2A\sin\left(\frac{N_{\mathrm{EO}}\Delta \beta }{2}\right)\sin\left(\frac{N_{\mathrm{EO}}\sum \beta }{2}\right)\sin\left(\phi \right) \end{array}$$

where $\Delta \beta =\beta_{2}-\beta_{1}$ and $\sum \beta =\beta_{2}+\beta_{1}$.

Furthermore, with the four sets of BTT signals in Table 1, six sets of blade vibration differences can be established as follows:

(20) $$\left\{\begin{array}{r} \begin{array}{r} \Delta y_{1}=2A\sin\left(\frac{N_{\mathrm{EO}}\Delta \beta_{1}}{2}\right)\cos\left(\frac{N_{\mathrm{EO}}\sum \beta_{1}}{2}\right)\cos\left(\phi \right)\\ \;-2A\sin\left(\frac{N_{\mathrm{EO}}\Delta \beta_{1}}{2}\right)\sin\left(\frac{N_{\mathrm{EO}}\sum \beta_{1}}{2}\right)\sin\left(\phi \right) \end{array}\\ \begin{array}{r} \Delta y_{2}=2A\sin\left(\frac{N_{\mathrm{EO}}\Delta \beta_{2}}{2}\right)\cos\left(\frac{N_{\mathrm{EO}}\sum \beta_{2}}{2}\right)\cos\left(\phi \right)\\ -2A\sin\left(\frac{N_{\mathrm{EO}}\Delta \beta_{2}}{2}\right)\sin\left(\frac{N_{\mathrm{EO}}\sum \beta_{2}}{2}\right)\sin\left(\phi \right) \end{array}\\ \vdots \\ \begin{array}{r} \Delta y_{6}=2A\sin\left(\frac{N_{\mathrm{EO}}\Delta \beta_{6}}{2}\right)\cos\left(\frac{N_{\mathrm{EO}}\sum \beta_{6}}{2}\right)\cos\left(\phi \right)\\ -2A\sin\left(\frac{N_{\mathrm{EO}}\Delta \beta_{6}}{2}\right)\sin\left(\frac{N_{\mathrm{EO}}\sum \beta_{6}}{2}\right)\sin\left(\phi \right) \end{array} \end{array}\right.$$

where the formula of Δβ and ∑β is listed in Table 2.

Equation (20) can be written in matrix form as follows:

(21) $$\Delta Y=\hat{B}\hat{X}$$

where

(22) $$\Delta Y={\left[\begin{array}{cccccc} \Delta y_{1} & \Delta y_{2} & \Delta y_{3} & \Delta y_{4} & \Delta y_{5} & \Delta y_{6} \end{array}\right]}^{T}$$

(23) $$\hat{B}=\left[\begin{array}{cc} 2\sin\left(\Delta {\hat{\beta }}_{1}\right)\cos\left(\sum {\hat{\beta }}_{1}\right) & -2\sin\left(\Delta {\hat{\beta }}_{1}\right)\sin\left(\sum {\hat{\beta }}_{1}\right)\\ 2\sin\left(\Delta {\hat{\beta }}_{2}\right)\cos\left(\sum {\hat{\beta }}_{2}\right) & -2\sin\left(\Delta {\hat{\beta }}_{2}\right)\sin\left(\sum {\hat{\beta }}_{2}\right)\\ \vdots & \vdots \\ 2\sin\left(\Delta {\hat{\beta }}_{6}\right)\cos\left(\sum {\hat{\beta }}_{6}\right) & -2\sin\left(\Delta {\hat{\beta }}_{6}\right)\sin\left(\sum {\hat{\beta }}_{6}\right) \end{array}\right]$$

(24) $$\hat{X}={\left[\begin{array}{cc} {\hat{x}}_{1} & {\hat{x}}_{2} \end{array}\right]}^{T}={\left[\begin{array}{cc} A\cos\left(\phi \right) & A\sin\left(\phi \right) \end{array}\right]}^{T}$$

where $\Delta \hat{\beta }=N_{\mathrm{EO}}\Delta \beta /2$ and $\sum \hat{\beta }=N_{\mathrm{EO}}\sum \beta /2$.

Equation (24) indicates that the vector $\hat{X}$ consists of the unknowns *A* and ϕ, while the coefficient vector $\hat{B}$ includes the unknown $N_{\mathrm{EO}}$. Similarly, the EO of blade synchronous vibration can be determined by the EO-search method, which is introduced as follows:

- The blade vibration difference ΔY was calculated by the BTT signals of one revolution and incorporating the estimated ${\tilde{N}}_{\mathrm{EO}}$ into the calculation coefficient matrix $\tilde{B}$. The corresponding vector $\tilde{X}$ was then solved using the least square method:

(25) $$\tilde{X}={\left({\tilde{B}}^{T}\tilde{B}\right)}^{-1}{\tilde{B}}^{T}\Delta Y$$

- Bring $\tilde{X}$ into (21), and define the residual difference between the fitted value and the actual value as follows:

(26) $$\tilde{E}\left({\tilde{N}}_{\mathrm{EO}}\right)=\tilde{B}\tilde{X}-\Delta Y$$

- The 2-Norm of residual $\tilde{E}\left({\tilde{N}}_{\mathrm{EO}}\right)$ was calculated to reflect the deviation of estimated ${\tilde{N}}_{\mathrm{EO}}$ from the real $N_{\mathrm{EO}}$:

(27) $$S\left({\tilde{N}}_{\mathrm{EO}}\right)={\left\|\tilde{E}\left({\tilde{N}}_{\mathrm{EO}}\right)\right\|}_{2}$$

In theory, when the estimated value equals the real value, there is $S\left({\tilde{N}}_{\mathrm{EO}}\right)=0$. Due to the measurement errors in the BTT technique, even when the EO is accurately estimated, $S\left({\tilde{N}}_{\mathrm{EO}}\right)$ tends to approach its minimum value. Furthermore, the vector $\tilde{X}$ can be solved by the least square method, and the parameters of blade synchronous vibration can be obtained. The blade vibration amplitude is calculated as follows:

(28) $$A=\sqrt{{\hat{x}}_{1}^{2}+{\hat{x}}_{2}^{2}}$$

The blade vibration phase is calculated as follows:

(29) $$\phi =\left\{\begin{array}{c} \begin{array}{cc} \arctan\left(\frac{{\hat{x}}_{2}}{{\hat{x}}_{1}}\right) & {\hat{x}}_{1}>0 \end{array}\\ \begin{array}{cc} \arctan\left(\frac{{\hat{x}}_{2}}{{\hat{x}}_{1}}\right)+\pi & {\hat{x}}_{1}<0 \end{array} \end{array}\right.$$

**Figure 1:**
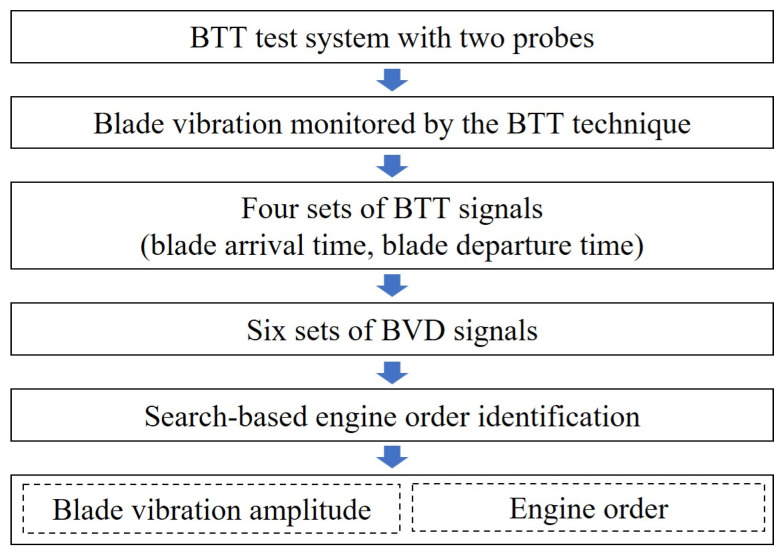
The flow chart of the BVD-CFF algorithm.

The flow chart of the BVD-CFF algorithm for identifying blade synchronous vibration parameters is displayed in Figure 1. The BVD-CFF algorithm utilizes the BTT signal to compute the BVD signals and, subsequently, employs the EO-search method to identify the blade synchronous vibration parameters. By enhancing the measurement accuracy of blade vibration, the BVD-CFF algorithm achieves high-precision identification of the blade synchronous vibration parameters.

## 4. Simulations

### 4.1. Numerical Simulation of Blade Synchronous Vibration

When the rotation speed passes through the synchronous resonance center of the blade, it induces blade resonance. Considering the single-mode resonance of the blade, the vibration response can be written as follows:

(30) $$y=\frac{A_{0}Q}{z}\frac{\eta \cos\left(N_{\mathrm{EO}}\beta +\phi \right)+\sin\left(N_{\mathrm{EO}}\beta +\phi \right)}{\left(1+\eta^{2}\right)}$$

where

(31) $$z=\frac{\omega }{\omega_{\mathrm{cen}}}$$

(32) $$Q=\frac{1}{2\xi }$$

(33) $$\eta =\frac{1}{2\xi }\frac{1-z^{2}}{z}$$

where $A_{0}$ is the static displacement of blade vibration caused by excitation force, $\omega_{\mathrm{cen}}$ is frequency of the blade synchronous resonance center, and ξ is the damping ratio.

**Figure 2:**
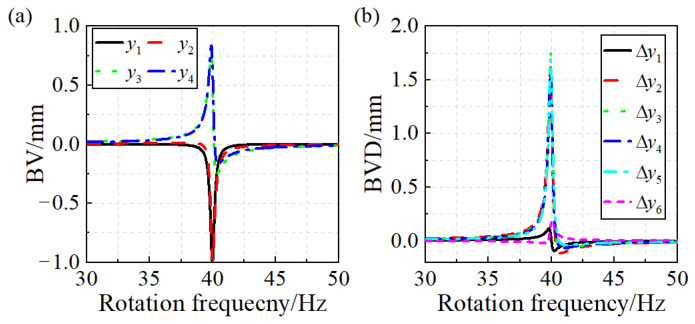
Simulation of blade synchronous vibration: (**a**) BV. (**b**) BVD.

The simulation of the blade synchronous vibration signal was conducted. The displacement caused by excitation force amplitude is $A_{0}=0.01\;\mathrm{mm}$, the blade resonance damping coefficient is ξ=0.005, the EO of blade vibration is $N_{\mathrm{EO}}=4$, the blade natural frequency is $f_{n}=\omega_{n}/2\pi =160\;\mathrm{Hz}$, the blade synchronous resonance center frequency is $f_{\mathrm{cen}}=\omega_{\mathrm{cen}}/2\pi =40\;\mathrm{Hz}$, the blade vibration phase is ϕ=π/3, the range of rotation frequency of rotor is 30~50 Hz, and the step size of simulation is set at increments of 0.01 Hz. During blade resonance conditions, the maximum value of blade vibration amplitude is $A_{\max}=A_{0}/2\xi =1\;\mathrm{mm}$. The parameters of two probes are $\beta =30^{\circ }$ and $\gamma =3^{\circ }$. The four sets of blade vibration signals and six sets of blade vibration difference signals are illustrated in Figure 2.

### 4.2. Discussion on the Feasibility and Superiority of the BVD-CFF Algorithm

**Figure 3:**
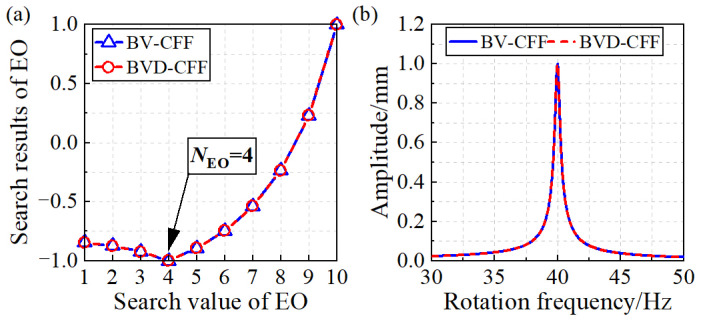
Identification results of blade synchronous vibration parameters: (**a**) Engine order of blade vibration. (**b**) Amplitude of blade vibration.

The CFF algorithm and the BVD-CFF algorithm are used to identify the parameters of simulation signals, respectively. As shown in Figure 3, the identification results of the EO and blade vibration amplitude are presented. Figure 3a shows that the EO of blade vibration obtained by the two algorithms is consistent with the simulation hypothesis. Furthermore, Figure 3b demonstrates that the two algorithms produce identical identification results for blade vibration amplitude. Hence, simulation studies validate the feasibility of the BVD-CFF algorithm.

**Figure 4:**
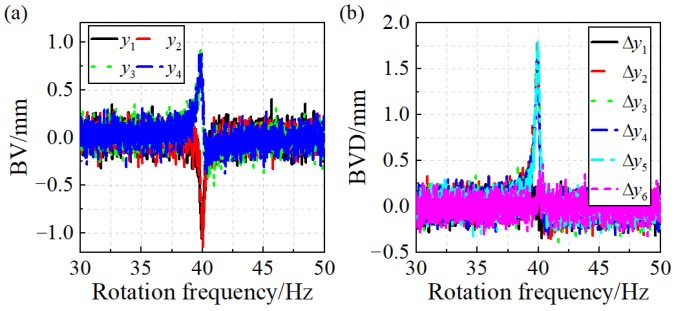
Simulation of the test errors of the blade synchronous vibration: (**a**) BV. (**b**) BVD.

Subsequently, accounting for the inherent uncertainties in practical applications, such as test errors, probe vibrations, and blade geometry inaccuracies, the noise simulation test errors are introduced into the blade vibration signals to evaluate the robustness of both algorithms. Gaussian white noise with an SNR ranging from 14 dB to 34 dB at a step size of 2 dB is added. Figure 4 illustrates the simulated blade vibration and blade vibration difference when the SNR is 20 dB.

**Figure 5:**
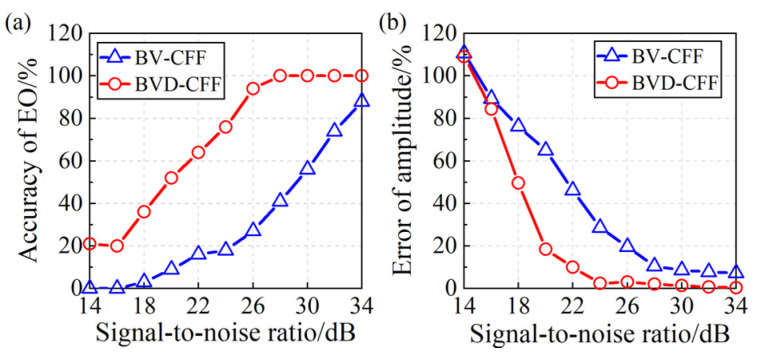
Research on the robustness of the BV-CFF algorithm and BVD-CFF algorithm: (**a**) Identification accuracy of EO. (**b**) Identification error of blade vibration amplitude.

Under different SNR conditions, two algorithms are used for the parameter identification in the simulated signals, including the EO and amplitude. To ensure eliminating the chance of simulation, the process (from signal simulation to parameter identification) is repeated 100 times under the identical SNR condition to ascertain the precision in identifying EO and amplitude. The identification results of blade synchronous vibration parameters are presented in Figure 5. The simulation results demonstrate the following: (1) The BVD-CFF algorithm exhibits excellent robustness in identifying EO, even in high SNR scenarios. (2) In terms of blade vibration amplitude identification, the BVD-CFF algorithm outperforms the BV-CFF algorithms with lower identification errors. Simulation and comparison with the BV-CFF algorithm validate the feasibility and superiority of the BVD-CFF algorithm.

## 5. Experiments

### 5.1. High-Speed Rotation Blade Vibration Test Rig

**Figure 6:**
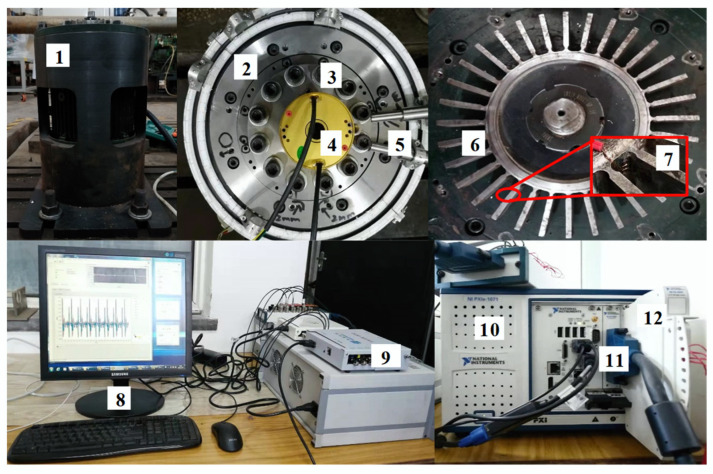
High-speed rotation blade vibration test rig: (1) Motor. (2) Protective cover. (3) Excitation magnet. (4) Slip ring. (5) Fiber optic probe. (6) Blade disk. (7) Strain gauge. (8) Computer. (9) Laser generator. (10) NI-PXI module. (11) Counter module. (12) Strain collection module.

The blade vibration experiments were carried out on a high-speed rotation blade vibration test rig based on the BTT technique, as shown in Figure 6, and the reliability of the proposed method in practical application is discussed. The test rig consists primarily of the rotation blade system, sensor system, signal acquisition system, and auxiliary system. The blade disk was mounted on the motor output shaft, with the motor rated at 12,000 rpm. The number of blades in the disk is 32, and the rotation diameter of the blade tip is 138 mm. The sensor system comprises optical fiber probes and strain gauges for measuring blade strain signals. The measurement results of blade strain can verify the accuracy of the BTT technique. For further details regarding the test rig, please refer to the literature [35].

### 5.2. Blade Synchronous Vibration Measurement

Six bolts with magnet excitation were uniformly arranged on the protective cover in a circular pattern to induce blade vibration. Additionally, two BTT probes were positioned along the circumference at an angle of 18.28°. The rotation speed range of the motor was set from 3120 rpm to 6120 rpm, with a running time of 300 s. Both the BTT signals and the strain signals were recorded, in which the sampling frequency of blade strain was set to 5000 Hz.

**Figure 7:**
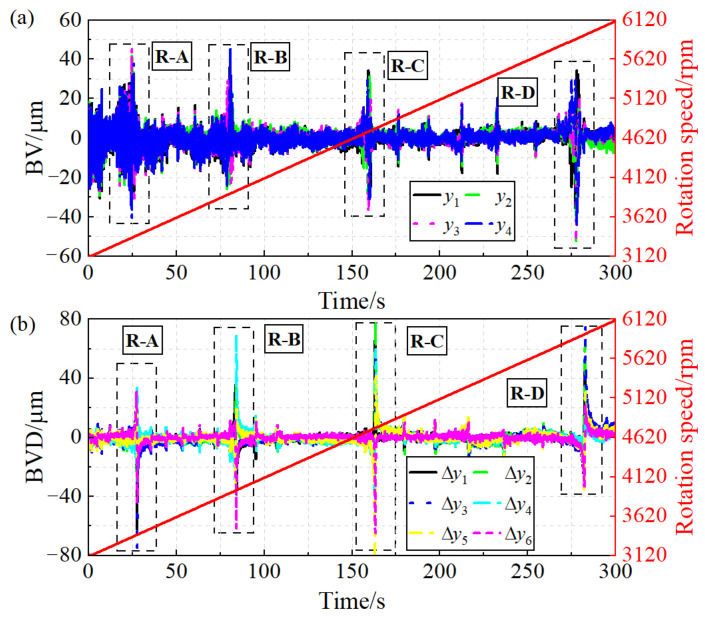
Blade synchronous vibration measurement results: (**a**) BV. (**b**) BVD.

According to the measurement method of blade vibration and blade vibration difference, four groups of blade vibration signals and six groups of blade vibration difference signals can be obtained through two probes. The vibration measurement results of the 16# blade are shown in Figure 7. The dashed line box in Figure 7 indicates that the rotation speed passes through the blade synchronous resonance center, resulting in a significant increase in blade vibration amplitude. The resonance regions are denoted as R-A, R-B, R-C, and R-D, respectively. Furthermore, the measurement results of blade vibration reveal that, compared to the blade vibration signals, the curves of blade vibration difference are smoother and exhibit higher measurement accuracy.

### 5.3. Blade Synchronous Vibration Parameter Identification

**Figure 8:**
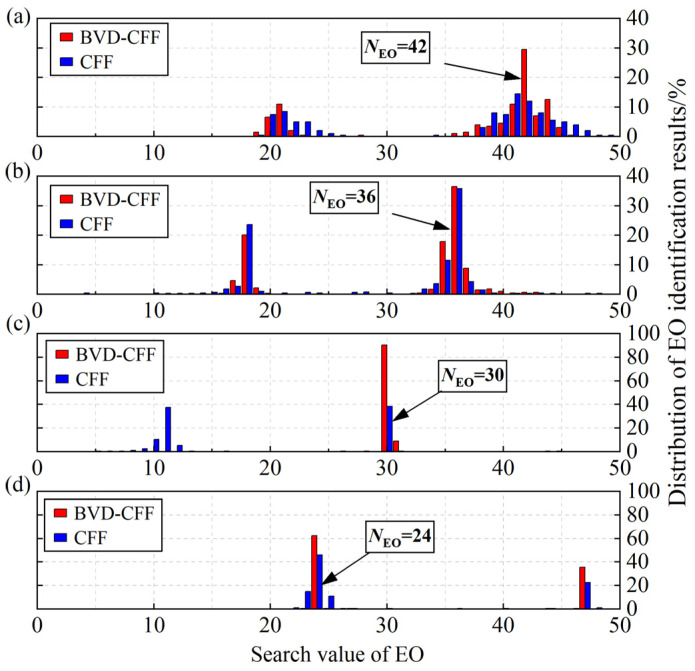
EO identification results of blade resonance: (**a**) R-A. (**b**) R-B. (**c**) R-C. (**d**) R-D.

**Figure 9:**
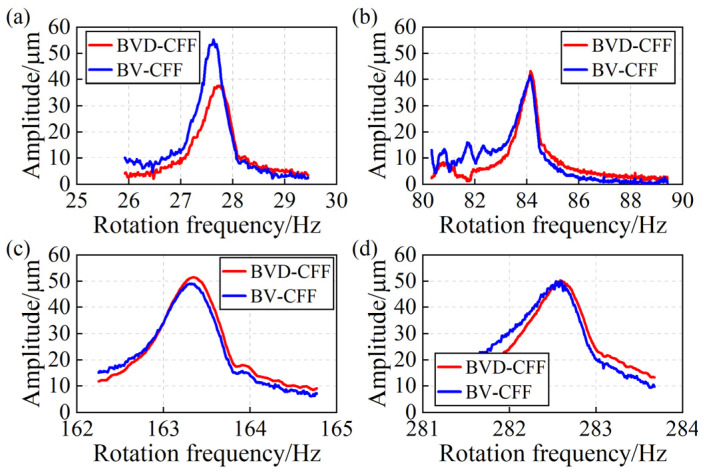
Amplitude identification results of blade resonance: (**a**) R-A. (**b**) R-B. (**c**) R-C. (**d**) R-D.

According to the blade vibration information, the BV-CFF algorithm and BVD-CFF algorithm are used to identify the parameters of blade synchronous vibration in the resonance region, respectively. Figure 8 illustrates the identification results of EO, while Figure 9 presents the identification results of blade vibration amplitude. Figure 8 shows that the BVD-CFF algorithm exhibits superior performance in EO identification, particularly within the low-speed resonance region characterized by a high SNR of blade vibration signals and limited accuracy of the BV-CFF algorithm for identification. Figure 9 shows that blade vibration amplitudes obtained by the two algorithms exhibit a high degree of consistency.

**Figure 10:**
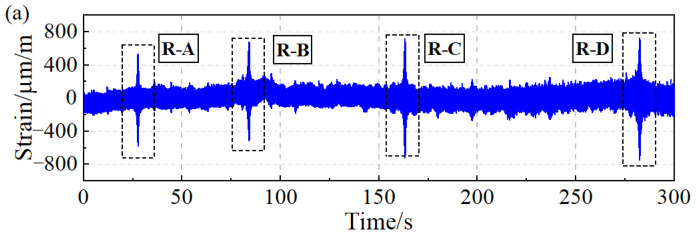

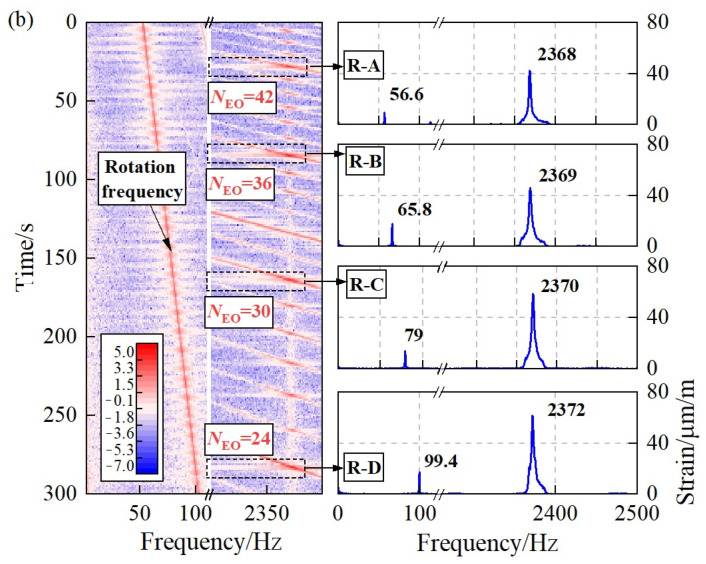
Analysis results of blade strain signals: (**a**) Time domain signals. (**b**) Time-frequency analysis results.

Through the time-frequency analysis of blade strain signals, the identification results of blade vibration can be verified. Figure 10 illustrates both the time domain signals and spectrum analysis results of the blade strain. It is evident that, within the resonance region, there is a significant increase in the blade strain, which aligns with the measured results of blade vibration. The spectrum analysis outcomes provide evidence for the accuracy of the BVD-CFF algorithm in EO identification.

## 6. Applications

### 6.1. Impeller Vibration Test of Centrifugal Compressor

**Figure 11:**
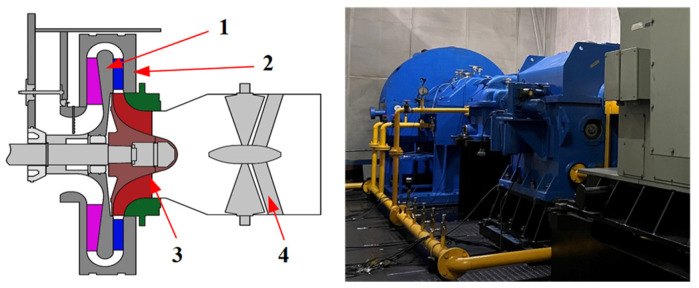
Φ800 centrifugal compressor test rig: (1) Return channel. (2) Diffuser. (3) Impeller. (4) Inlet guide.

The method presented in this paper is also applicable to the vibration measurement and analysis of the impeller of a centrifugal compressor. In this study, authors conducted vibration tests on the Φ800 centrifugal compressor test rig to verify the blade vibration testing and parameter identification methods. The structure diagram and physical diagram of the centrifugal compressor test rig are depicted in Figure 11, comprising primarily components such as the inlet guide vane, centrifugal impeller, diffuser, and return channel, among others. The inlet guide vane of the centrifugal compressor is positioned within the intake pipe to effectively manipulate the axial airflow angle. The centrifugal impeller boasts a total of 19 blades, while its inlet directly interfaces with the ambient atmosphere, utilizing it as the working medium gas for compression purposes. Moreover, the outlet pipe is oriented vertically in an open-loop experimental system configuration. Notably, the driving motor powering this centrifugal compressor exhibits a robust capacity of 2.1 MW, enabling the rated speed of the impeller to reach 9000 rpm.

**Figure 12:**
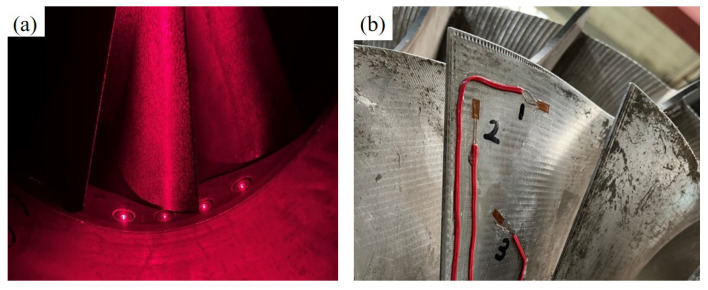
Probe layout for impeller vibration test of centrifugal compressor: (**a**) Fiber optic probe. (**b**) Strain gauge.

The vibration measurement and analysis of the centrifugal compressor impeller are conducted using the BTT technique and the SG method. Two optical fiber probes were installed on the inlet ring of the centrifugal compressor, positioned at an installation angle of 10°. Additionally, strain gauges were affixed to the impeller blade surface to measure vibration stress, thereby validating the accuracy of the BTT test results. The probe layout for impeller vibration testing in a centrifugal compressor is illustrated in Figure 12.

**Figure 13:**
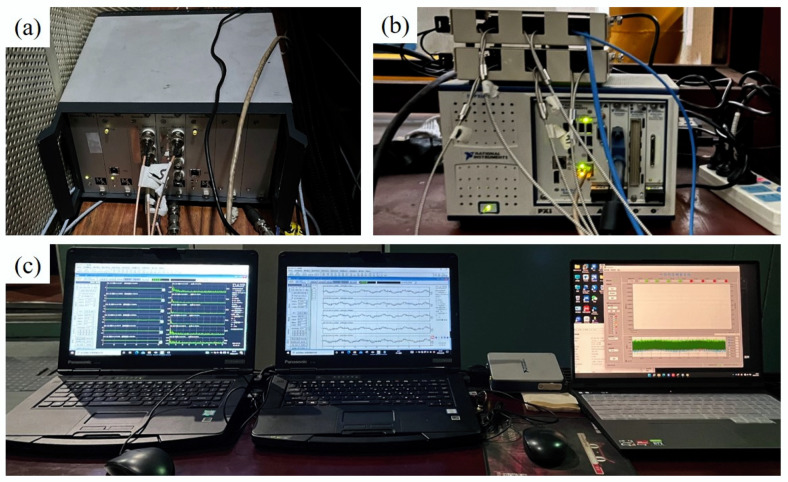
Impeller vibration test system: (**a**) Strain signal acquisition module. (**b**) Blade tip timing acquisition module. (**c**) Blade vibration monitoring system.

The impeller vibration test system of the centrifugal compressor consists of the strain test system and the BTT test system. The strain signal transmission utilizes the telemetry system provided by MANNER, a German company. The strain signals are transmitted to the DASP acquisition system of the computer through the NI-9231 acquisition card, and the sampling frequency of the strain signals is 10,240 Hz. The BTT test system adopts the laser generator from the HOOD company in the United States. As the blade passes through the probe, square wave signals are generated and transmitted to the PCI-6602 acquisition card by the probe, and the arrival time and departure time of the blade are obtained. Figure 13 illustrates the impeller vibration test system.

The impeller vibration experiments of the centrifugal compressor were conducted under varying rotation speed conditions. In consideration of operational safety, the inlet guide vane angle of the compressor was adjusted to 90°, while the throttle valve remained fully open to ensure the maximum flow state. Subsequently, after achieving a steady operation of the centrifugal compressor, the rotation speed of test rig was manually adjusted from 2500 rpm to 5000 rpm.

### 6.2. Impeller Vibration Measurement Results of Centrifugal Compressor

**Figure 14:**
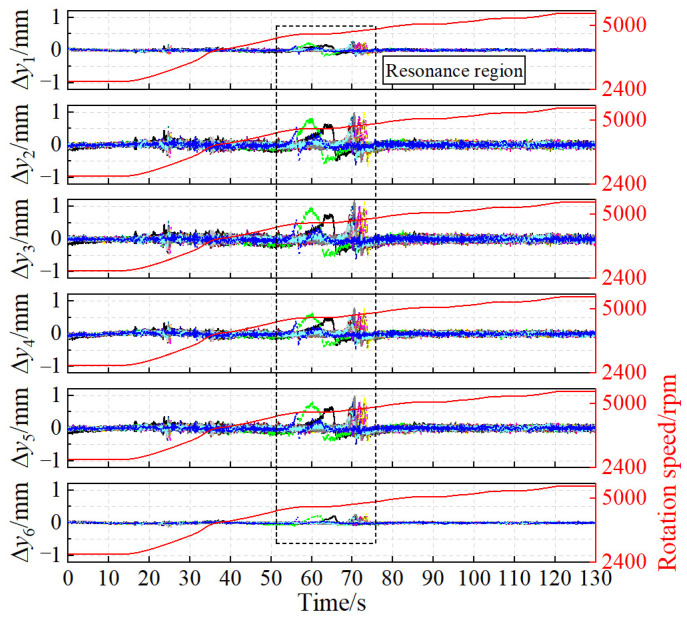
Calculation results of blade vibration difference in centrifugal impeller.

According to the blade vibration difference theory, six groups of blade vibration difference signals can be obtained from the four sets of BTT signals obtained by the two probes. Figure 14 presents the calculation results of the vibration differences of 19 blades. The black dashed line in Figure 14 shows that the blade vibration amplitude increases significantly, indicating that the rotation speed passes through the blade synchronous resonance center.

**Figure 15:**
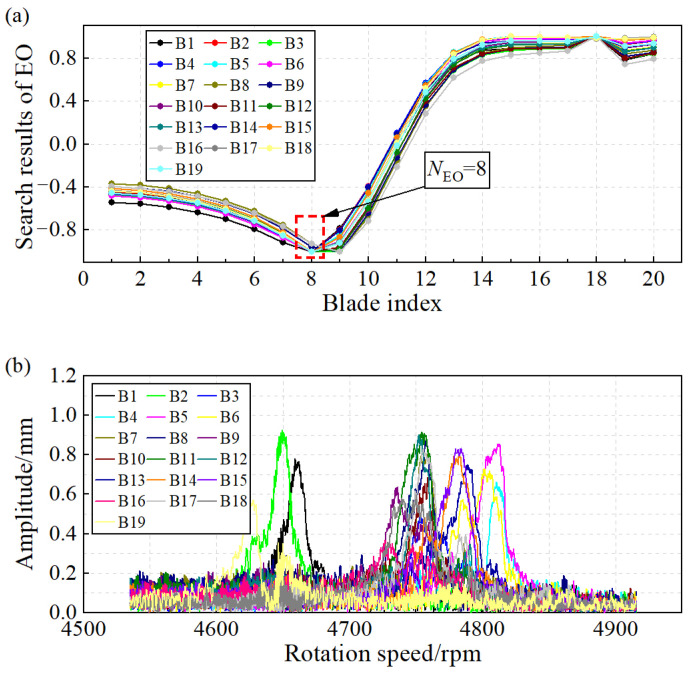
Identification results of blade synchronous vibration parameters of centrifugal impeller: (**a**) EO of blade vibration. (**b**) Amplitude of blade vibration.

The BVD-CFF algorithm was utilized to analyze the blade synchronous vibration signals, and the identification results of blade synchronous vibration parameters are presented in Figure 15. Figure 15a illustrates that the EO is eight at the synchronous resonance center, while Figure 15b displays the identification results of blade vibration amplitude.

**Figure 16:**
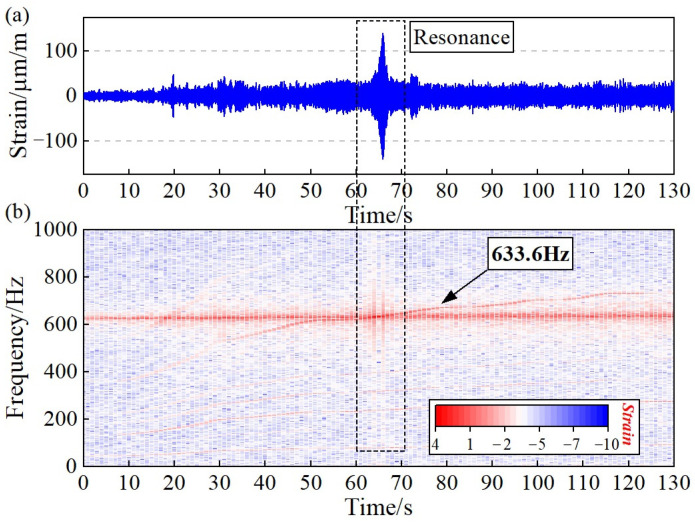
Blade strain signal analysis results of centrifugal impeller: (**a**) Time domain signal. (**b**) Time-frequency analysis results.

Additionally, Figure 16 demonstrates the analysis findings of blade strain signals, indicating that the rotation frequency is 78.8 Hz when the vibration frequency of the blade reaches resonance at 633.6 Hz. Thus, it can be inferred that the EO of the blade is 633.6/78.8=8.04≈8. Therefore, these results confirm that the BVD-CFF algorithm has high reliability for practical engineering applications.

## 7. Conclusions

The BVD theory provides an effective solution for high-precision measurements of BTT technique. In this paper, the traditional CFF algorithm is optimized, four sets of BTT signals are obtained by two probes, six sets of blade vibration difference signals are established, and the BVD-CFF algorithm is proposed. Simulations and experiments demonstrate that the BVD-CFF algorithm exhibits superior anti-noise performance and yields identification results for blade vibration parameters that are largely consistent with those obtained through the SG method. Furthermore, the research on the impeller vibration of the centrifugal compressor confirms that the BVD-CFF algorithm accurately identifies blade synchronous vibration parameters, thereby validating its feasibility and reliability for practical engineering applications. The research findings presented in this paper possess significant guiding implications and reference values for the blade vibration monitoring and blade operational state evaluation in major equipment. Nevertheless, the algorithm proposed in this article also has limitations, and it is only applicable for identifying synchronous resonance parameters of blades under varying speed conditions. Additionally, the identification of blade asynchronous vibration parameters remains a crucial aspect in the BTT technique, necessitating further exploration in future work.

## Figures and Tables

**Table 1:** The BTT signals obtained by two probes.

Probe Index	*p* = 1		*p* = 2	
Signal Type	Arrival Time	Departure Time	Arrival Time	Departure Time
Signal	$t_{\left(i,1\right)}^{ToA}$	$t_{\left(i,1\right)}^{ToD}$	$t_{\left(i,2\right)}^{ToA}$	$t_{\left(i,2\right)}^{ToD}$
Installation angle	0	γ	β	β+γ

**Table 2:** The formula of Δβ and ∑β.

Subscript	1	2	3	4	5	6
Δβ	γ	β	β+γ	β−γ	β	γ
∑β	γ	β	β+γ	β+γ	β+2γ	2β+γ

## Data Availability

The data that support the findings of this study are available on request from the corresponding author. The data are not publicly available due to privacy or ethical restrictions.

## Nomenclature

BTT	Blade tip timing
BV	Blade vibration
BVD	Blade vibration difference
BV-CFF	Blade vibration-based circumferential Fourier fitting
BVD-CFF	Blade vibration difference-based circumferential Fourier fitting
EO	Engine order
SG	Strain gauge
*A*	Amplitude of blade vibration
*A*_0_	Static displacement of blade vibration caused by excitation force
*C*	Blade vibration offset
*i*	Blade index
*n*	Number of revolutions
*N*_b_	Number of blades
*N*_EO_	Engine order of blade vibration
*p*	Probe index
*R*	Blade radius
*t*	Actual arrival time of blade
*y*	Blade vibration
Δy	Blade vibration difference
β	Installation angle of the probe
τ	Expected arrival time of the blade
ϕ	Phase of blade vibration
ω	Rotation speed
$\omega_{b}$	Frequency of blade vibration
$\omega_{\mathrm{cen}}$	Blade synchronous resonance center
ξ	Damping ratio
γ	Rotation radian when the blade passes the probe
